# Comment on: Risk factors associated with multidrug-resistant tuberculosis in areas with a moderate tuberculosis burden

**DOI:** 10.1093/inthealth/ihae060

**Published:** 2024-09-25

**Authors:** John Patrick C Toledo

**Affiliations:** Department of Theology and Religious Education, De La Salle University, 2401 Taft Avenue, 1004 Manila, Philippines

To The Editor,

I am writing to share my insights on recent research published on 14 June 2024, entitled ‘Risk factors associated with multidrug-resistant tuberculosis in areas with a moderate tuberculosis burden’ by Mansoori et al.,^1^ and to look at its contribution, especially in the context of public health in the Philippines.

This study identifies several key risk factors contributing to the development of multidrug-resistant TB (MDR-TB), which include previous TB treatment, poor adherence to TB medication, HIV coinfection and socioeconomic factors such as poverty and lack of access to healthcare. The research also highlights the significance of early detection and appropriate treatment regimens to prevent the spread of MDR-TB.

By analyzing data from regions with a moderate TB burden, your study provides a comprehensive understanding of the local epidemiology and the challenges faced in managing and controlling MDR-TB. The contributions and advantages of the paper are: (i) targeted interventions: the identification of specific risk factors allows for the design of targeted interventions, which can be more effective in preventing and managing MDR-TB; (ii) resource allocation: understanding the socioeconomic and healthcare-related factors contributing to MDR-TB can guide the allocation of resources to areas and populations most in need; and (iii) policy development: your findings can inform policymakers in the development of strategies and policies aimed at reducing the incidence of MDR-TB.

According to the WHO, a total of 1.3 million people died from TB in 2022 (including 167 000 people with HIV). Worldwide, TB is the second leading infectious killer after coronavirus disease 2019 (COVID-19) (above HIV and AIDS).^2^ As a result, TB is now the second deadliest infectious disease in the world, killing more people than HIV/AIDS and other illnesses combined. It is only surpassed by COVID-19. This is why TB control efforts can be made more effective by putting into practice specific interventions based on the risk factors that have been identified. Drug resistance can be considerably inhibited, for example, by providing patient education and support to ensure improved adherence to TB medication. Furthermore, addressing socioeconomic determinants of health may have a more extensive and long-lasting effect on TB control.

Mansoori et al. provide important information on the drug resistance pattern of *Mycobacterium tuberculosis* strains.^1^ In conclusion, the paper provides valuable insights that can develop the understanding and management of MDR-TB. Its application, especially for those countries that have the highest numbers of TB cases, could lead to significant developments in public health awareness.

## Data Availability

The data that support the findings of this study are available from the WHO.
